# Social value orientation and college students’ aggressive behaviors: the chain mediating effect of perspective taking and general trust

**DOI:** 10.3389/fpsyg.2026.1907962

**Published:** 2026-08-18

**Authors:** Chunhui Qi, Zhen Zhang

**Affiliations:** Faculty of Education, Henan Normal University, Xinxiang, China

**Keywords:** aggressive behavior, college student, general trust, perspective taking, social value orientation

## Abstract

To explore the chain mediating effect of perspective taking and general trust between social value orientation and college students’ aggressive behavior, and to provide scientific evidence for promoting college students’ mental health and reducing campus violence. The current study adopts a combination of cluster sampling and convenience sampling methods, selecting 1,994 students from 6 universities in Guangdong, Fujian, Jiangsu, Henan, Tianjin, and Xizang provinces. The measurements include the Social Value Orientation Slider Measure (SVO-SM), the Perspective Taking sub-scale of the Interpersonal Reactivity Index (IRI-PT), the General Trust Scale (GTS), and the Short-Form Buss-Perry Aggression Questionnaire (BPAQ-SF). The results show that the scores of social value orientation, perspective taking, and general trust are all positively correlated with each other, and all three are negatively correlated with aggressive behavior. The mediation effect analysis indicates that social value orientation can predict aggressive behavior through perspective taking, general trust, and the mediating chain of the two, with the mediation effects of the three paths being 9.017%, 4.378%, and 0.947% respectively. This suggests that perspective taking and general trust of college students have multiple mediating effects between social value orientation and aggressive behavior. The present associations provide preliminary implications for college education practices centered on prosocial skill development, which tend to correspond to a lower prevalence of campus bullying and aggressive conduct.

## 1 Introduction

Aggressive behavior refers to any pattern of behavior that attempts to harm someone and where the other party tries to avoid such harm (Vega et al., 2022). It is widespread in the biological world and takes on more complex forms in primates. In human society, aggression is an important indicator for measuring an individual’s social adaptability and has long been a subject of extensive research (Allen et al., 2018; Gil-Juliá et al., 2025). School violence and aggressive behavior are global mental health and public health issues (Kowalski et al., 2014; Devries et al., 2022; Li and Du, 2024). Chronic engagement in bullying and aggressive acts correlates with higher rates of substance misuse, criminal conduct, and violent outcomes (Basto-Pereira et al., 2022; Blattman et al., 2023; Şeker and Akgür, 2025). Victims of bullying are positively correlated with low self-esteem and low self-worth, and are prone to psychological problems such as anxiety and depression, and even self-harm and suicidal behavior (Alini et al., 2025; Kennedy et al., 2025; Serafini et al., 2023). School aggression and violence have become urgent theoretical and practical issues that schools, society, and the government are paying close attention to (Li et al., 2022; McMahon et al., 2024).

Scholars from different disciplines have focused on exploring various protective factors and measures for effectively preventing, weakening, or intervening in aggressive behaviors among college students (Alini et al., 2025; Blattman et al., 2023; McMahon et al., 2024). The I3 theory of aggression posits that instigation, impellance, and inhibition are three independent cognitive processes that affect aggressive behavior (Finkel and Hall, 2018). Instigation refers to the social dynamic separation between the instigator and the potential victim, often leading to a strong desire for aggression; impellance refers to the process of strengthening the desire for aggression; inhibition refers to the process of weakening or overcoming the desire for aggression. Many psychological factors influencing aggressive behavior can be classified into these processes (Doménech et al., 2025), among which social value orientation is an important influencing factor in the inhibition process (Murphy et al., 2011; Pletzer et al., 2018). As a prosocial personality trait, the cognitive mechanism by which social value orientation weakens aggressive behavior is not yet clear. Therefore, this study aims to examine the impact of college students’ social value orientation on aggressive behavior and test the sequential mediating role of perspective taking and general trust.

### 1.1 Literature review and research hypotheses

#### 1.1.1 Social value orientation and aggressive behavior

Social value orientation (SVO) refers to the specific preferences of an individual for the allocation of resources between oneself and others in a dependent situation, and it is divided into egoistic individuals who pursue self-interest and prosocial individuals who focus on the interests of others. It is a key motivational factor explaining cooperation and conflict decisions (Zhang et al., 2014, 2015, 2025). Social value orientation has strong stability across time and across contexts (Murphy and Ackermann, 2014), and is automatically manifested in an individual’s behavioral patterns (Cornelissen et al., 2011), enabling prosocial individuals to exhibit stronger reciprocity expectations and cooperative behaviors (Pletzer et al., 2018). Compared to prosocial individuals, egoistic individuals are more concerned about their own interests, more suspicious of others’ behavioral intentions, have a strong tendency for hostile attribution, and exhibit more aggressive behaviors. In the dictator game task assessing proactive aggression, empirical studies have found that prosocial individuals exhibit lower aggressive behaviors than egoistic individuals (Cornelissen et al., 2011; Hilbig and Zettler, 2009). At the same time, in the ultimatum game task evaluating reactive aggression, prosocial individuals are also more willing to accept unfair proposals than egoistic individuals (Karagonlar and Kuhlman, 2013). More importantly, with the rise of positive psychology, researchers have gradually focused on the correlational linkage between prosocial skills development and lower aggression (Jeuken et al., 2015). Greitemeyer and Mügge (2014) conducted a meta-analysis and found that exposure to prosocial games correlates with lower aggression and related outcomes, alongside higher prosocial behavior. Based on this, we propose research hypothesis 1: Social value orientation can negatively predict aggressive behavior.

#### 1.1.2 Perspective taking as a mediator

As an essential psychological quality for interpersonal interaction, perspective taking is an individual’s ability to grasp others’ viewpoints from their perspectives, which is a cognitive strategy and ability of a prosocial tendency (Sassenrath et al., 2022). It is closely related to social value orientation and aggressive behavior. On one hand, there are significant differences in the understanding of others’ social motivations between prosocial individuals and egoistic individuals. Prosocial individuals tend to view issues from the other person’s perspective and have stronger perspective taking, while egoistic individuals are more selfish and have weaker perspective taking (Declerck and Bogaert, 2008; Derks et al., 2015; Dong et al., 2018). On the other hand, a large number of empirical studies have found that perspective taking can significantly negatively predict the aggressive tendencies of adolescents (Kahhale et al., 2024; Pozzoli et al., 2017; Deng et al., 2025; Romero et al., 2024). Systematic reviews and meta-analyses also indicate that perspective taking can effectively inhibit the aggressive behavior of adolescents (Carmona-Cardona et al., 2024; Hall et al., 2021). Therefore, we propose research hypothesis 2: Social value orientation can predict aggressive behavior through perspective taking.

#### 1.1.3 General trust as a mediator

General trust refers to an individual’s willingness, in situations of information scarcity, to voluntarily entrust social resources to others based on expectations of goodwill toward them (Hancock et al., 2023). Given the risks and uncertainties associated with trust, such as the potential for deception and betrayal, social value orientations can effectively enhance individuals’ levels of trust (Derks et al., 2014; Kanagaretnam et al., 2009), leading to greater tendencies toward trust. Derks et al. (2014) examined the effects of gender and value orientation on general trust among adolescents, finding that prosocial individuals exhibited more trust and reciprocal behaviors than those oriented toward self-interest. Furthermore, general trust is tend to accompany fewer individual involvement in school violence and aggressive behaviors through multiple pathways (Rotenberg et al., 2021; Rotenberg and Fonseca, 2024; Yang et al., 2025). Based on longitudinal data from 1,148 Chinese college students, Yang et al. (2025) found that higher general trust correlates with a less steep growth trajectory of social aggression, such that the higher the overall trust level among students, the lower their level of social aggression, and vice versa. Therefore, we propose Hypothesis 3: General trust may mediate the relationship of social value orientation on aggression.

#### 1.1.4 Perspective taking and general trust

There is also a close relationship between perspective taking and general trust, with perspective taking playing a crucial role in the emergence and development of general trust (Evans and Krueger, 2011). Individuals with stronger perspective taking abilities are better at viewing issues from others’ viewpoints and tend to have higher levels of trust (Krabbendam et al., 2024). Moreover, the enhancement of perspective taking ability associated with age progression during adolescence is an important factor contributing to increased trust among adolescents (Fett et al., 2014). Additionally, numerous experimental studies have found that manipulations and training in perspective taking can significantly boost people’s general trust (Erle et al., 2018; Kong et al., 2023). More importantly, Qi et al. (2019) discovered that the chain involving perspective taking and general trust mediates the relationship between self-control and aggressive behavior in college students. Therefore, we propose Hypothesis 4: the chain of perspective taking and general trust may also serve as a potential pathway through which social value orientation suppresses aggressive behavior.

**FIGURE 1:**
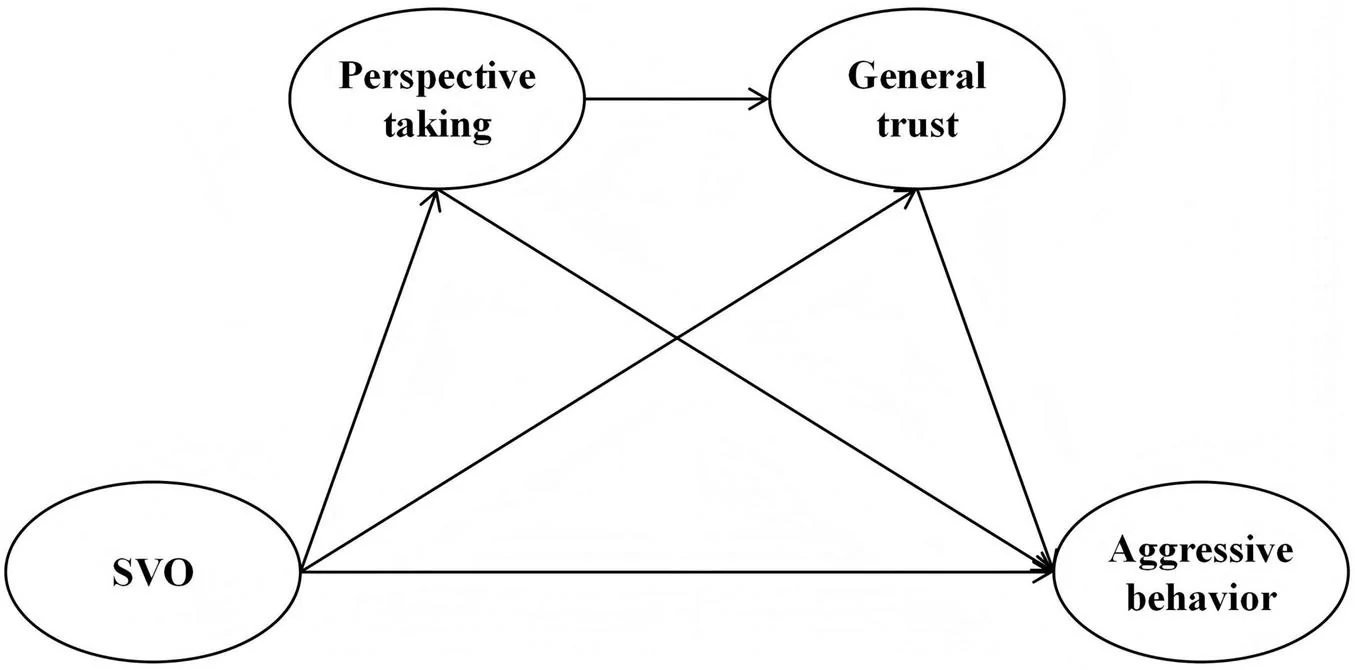
Research model.

In summary, this study examines the mechanism through which social value orientation attenuates aggressive behavior among college students, with perspective taking and general trust serving as sequential mediators. By integrating these constructs into a coherent theoretical framework, the study aims to advance understanding of prosocial motivational pathways and to inform evidence-based prevention and intervention strategies targeting aggression in higher education settings. The proposed theoretical model is presented in Figure 1.

## 2 Method

### 2.1 Participants and procedure

Six universities in Guangdong, Fujian, Jiangsu, Henan, Tianjin and Xizang were selected as the survey subjects. A combined method of cluster sampling and convenience sampling was adopted to distribute a total of 2,100 questionnaires. After eliminating the invalid ones, 1,994 valid questionnaires were retrieved, with a valid recovery rate of 94.95%. Among them, there were 720 students from Guangdong, 98 from Fujian, 361 from Jiangsu, 241 from Henan, 186 from Tianjin, and 388 from Xizang; 653 freshmen, 911 sophomores, and 430 juniors; 1,339 females and 655 males; 518 only children and 1,476 non-only children; 1,199 students majoring in science and technology, and 795 students majoring in liberal arts. The participants were aged between 16 and 25, with an average age of 19.68 years.

Data collection was carried out by a graduate researcher who had undergone thorough, rigorous training and administered psychometrically validated instruments following well-established, standardized protocols. Prior to each session, participants were kindly provided with clear, comprehensive, and scripted procedural instructions to support consistent understanding and thoughtful task engagement. All assessments took place in quiet, carefully controlled environments designed to minimize distractions, and responses were collected anonymously and securely. To help safeguard the integrity of the data and to respectfully support both participant autonomy and honest responding, the study emphasized fully voluntary participation and strict confidentiality. No personal identifiers were collected at any stage. Participants were verbally and in writing reaffirmed that withdrawal at any stage would entail no academic, social, or procedural consequences. Each testing session was precisely timed to 20 min to maintain procedural consistency across participants. Ethical approval for this study was granted by the Ethics Committee of the Faculty of Education at Henan Normal University, ensuring compliance with ethical research standards.

### 2.2 Measures

#### 2.2.1 Social value orientation

The Chinese adaptation of the Social Value Orientation Slider Measure (SVO-SM) was employed to assess participants’ social value orientation. Originally developed by Murphy et al. (2011) and subsequently adapted for Chinese populations by Zhang et al. (2015), this instrument comprises six dichotomous allocation items, each presenting nine paired resource distribution options. Respondents select the option that best reflects their preference, and a continuous value orientation angle ranging from self-interest to other-regarding motivation is computed based on their pattern of choices. A higher angular value indicates a stronger orientation toward maximizing others’ outcomes relative to one’s own.

#### 2.2.2 Perspective taking

The Perspective Taking sub-scale of the Interpersonal Reactivity Index (IRI-PT) was employed to evaluate participants’ perspective taking ability. Originally developed by Davis (1983) and subsequently revised by Zhang et al. (2010), the IRI-PT comprises five Likert-type items (e.g., “Sometimes I look at problems from my friend’s perspective to better understand them”), each rated on a 5-point scale ranging from 1 (strongly disagree) to 5 (strongly agree). Total scores are computed by summing item responses, with higher scores indicating greater perspective taking proficiency. In the present study, the internal consistency of the IRI-PT, as indexed by Cronbach’s α, was 0.71.

#### 2.2.3 General trust

The General Trust Scale (GTS), originally developed by Yamagishi et al. (2015) and subsequently revised by Zhang et al. (2019), comprises nine Likert-type items assessing generalized trust in others (e.g., “Most people are trustworthy”). Responses are rated on a 7-point scale, anchored at 1 (“completely inappropriate”) and 7 (“completely appropriate”). In the present study, the GTS demonstrated satisfactory internal consistency, with Cronbach’s α = 0.83.

#### 2.2.4 Aggressive behavior

The Short-Form Buss-Perry Aggression Questionnaire (BPAQ), originally developed by Buss and Perry (1992) and subsequently adapted into a shortened form by Diamond and Magaletta (2006), was further culturally validated and refined by Zhang et al. (2009) for use in the present study. This 12-item instrument assesses four empirically derived dimensions of aggression: physical aggression, verbal aggression, anger, and hostility. Sample items include “If I get angry at someone, I might hit him or her.” Responses are rated on a 5-point Likert scale ranging from 1 (strongly disagree) to 5 (strongly agree), with higher total scores indicating greater self-reported aggression. Internal consistency for the scale in the current sample was satisfactory, as evidenced by a Cronbach’s α coefficient of 0.81.

### 2.3 Data analysis

Descriptive statistical analysis, correlation analysis, and tests for common method bias were conducted using SPSS 19.0. Specifically, Harman’s single-factor test was performed to assess potential common method variance. Bivariate Pearson correlation coefficients were computed to examine the linear relationships among all study variables. To test the hypothesized multiple mediation model, Model 6 of Hayes (2013) PROCESS macro for SPSS was employed, with bootstrapping based on 5,000 resamples to generate 95% bias-corrected confidence intervals for indirect effects. The significance level α was set at 0.05.

## 3 Results

### 3.1 Assessment of common method variance

Given cross-sectional self-report data, we tested common method bias (CMB) using two methods (Podsakoff et al., 2012, 2024).

First, unrotated EFA for Harman’s single-factor test showed the first factor explained under 40% total variance. This is only a preliminary screen: the 40% cutoff lacks statistical basis and cannot separate trait, method and random error, making it insensitive to mild CMB.

We further performed nested CFA with robust maximum likelihood via the unmeasured latent method construct (ULMC) approach. The baseline model included three correlated substantive factors. The augmented ULMC model added a global method factor loading all items, constrained orthogonal to trait constructs to isolate method variance. Model fit statistics and comparative changes are reported below:

Baseline: χ^2^ = 3486.21, df = 649, CFI = 0.912, RMSEA = 0.047

ULMC model: χ^2^ = 2788.51, df = 623, CFI = 0.948, RMSEA = 0.041

Changes: Δχ^2^ = 697.70, Δdf = 26, ΔCFI = 0.036, ΔRMSEA = −0.006

The significant Δχ^2^ is expected given our large sample (*N* = 1,994), as chi-square tests overstate minor fit gains. Most importantly, the maximum shift in latent construct correlations was merely 0.011, well below the 0.20 threshold for severe CMB.

Triangulating both analyses, severe common method bias is absent; self-report method variance does not meaningfully bias focal construct relationships.

### 3.2 Descriptive statistics and correlation analysis

The analytical results indicate statistically significant positive associations between social value orientation, perspective taking, and general trust (*r* = 0.15, *p* < 0.001; *r* = 0.17, *p* < 0.001; *r* = 0.23, *p* < 0.001, respectively), as well as statistically significant negative associations between each of these three constructs and aggressive behavior (*r* = –0.32, *p* < 0.001; *r* = –0.26, *p* < 0.001; *r* = –0.19, *p* < 0.001, respectively). Further details are presented in Table 1.

**TABLE 1:** Descriptive statistics and correlations matrix of all variables (*n* = 1994).

Variables	*M ± SD*	1	2	3	4
1. Social value orientation	31.05 ± 10.90	–	–	–	–
2. Perspective taking	3.51 ± 0.61	0.15***			
3. General trust	3.39 ± 0.68	0.17***	0.23***		
4. Aggressive behavior	2.33 ± 0.58	–0.32***	–0.26***	–0.19***	

***p < 0.001.

### 3.3 Chain mediation effect analysis

**FIGURE 2:**
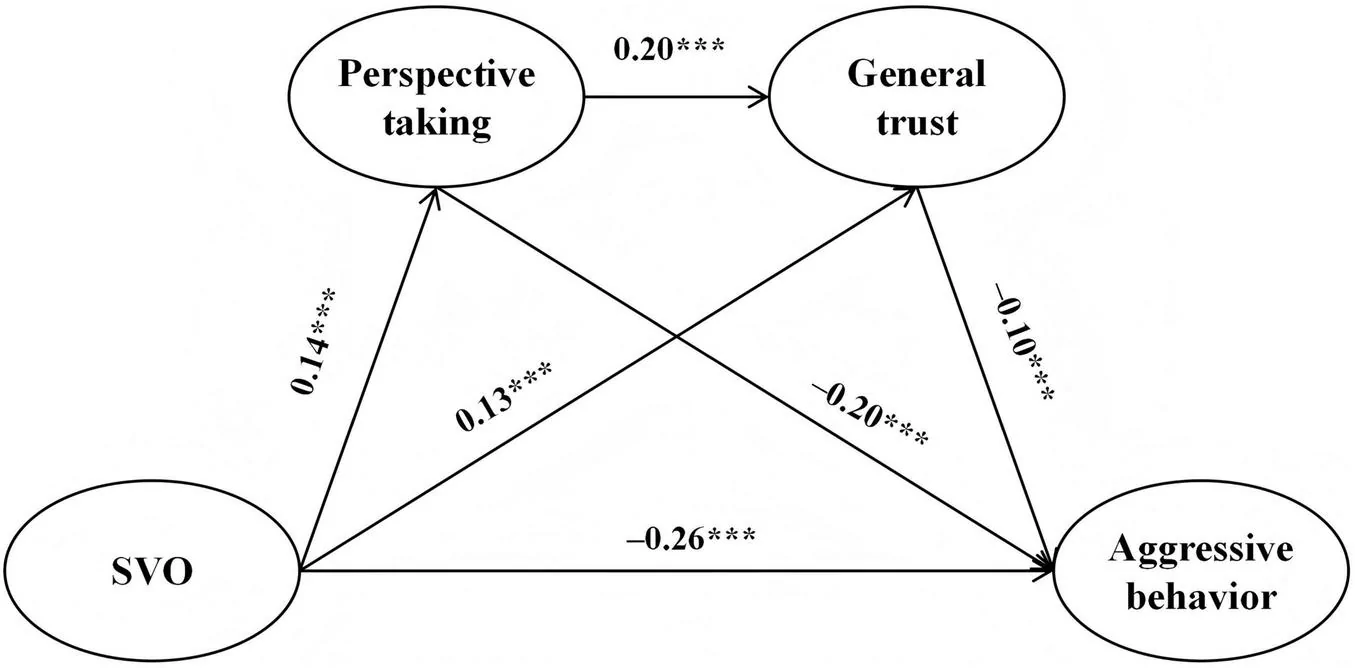
Chain mediation model of perspective taking and general trust in social value orientation and aggressive behavior. ***p < 0.001.

After controlling for covariates (gender, age, grade level, only-child status, academic major, and province of residence), social value orientation emerged as a significant negative predictor of aggressive behavior (β = −0.31, *p* < 0.001) and a significant positive predictor of perspective taking (β = 0.14, *p* < 0.001). Furthermore, both social value orientation and perspective taking significantly and positively predicted general trust (β = 0.13, *p* < 0.001; β = 0.20, *p* < 0.001, respectively). In the full model predicting aggressive behavior, social value orientation, perspective taking, and general trust were each significantly and negatively associated with aggressive behavior (β = −0.26, *p* < 0.001; β = −0.20, *p* < 0.001; β = −0.10, *p* < 0.001, respectively; see Table 2). These findings indicate that perspective taking and general trust jointly mediate the relationship between social value orientation and aggressive behavior, with evidence supporting a partial mediation effect. The path coefficient results are shown in Figure 2.

**TABLE 2:** Regression analysis among variables in the chain mediation model.

Regression equation		Overall fitting index			Regression coefficient		
Result variable	Prediction variable	*R*	*R* ^2^	*F*	β	*t*	95% CI
Aggressive behavior	Social value orientation	0.34	0.11	36.81***	–0.31	–14.44***	[−-0.35 –0.26]
Perspective taking	Social value orientation	0.18	0.03	9.39***	0.14	6.32***	[0.10 0.18]
General trust	Social value orientation	0.41	0.17	51.14***	0.13	6.27***	[0.09 0.17]
	Perspective taking				0.20	9.41***	[0.15 0.24]
Aggressive behavior	Social value orientation	0.41	0.17	45.00***	–0.26	–14.81***	[−-0.30 –0.22]
	Perspective taking				–0.20	–9.26***	[−-0.24 –0.16]
	General trust				–0.10	–4.64***	[−-0.15 –0.06]

All variables in the model are brought back into the equation after standardized processing,

***p < 0.001.

Table 3 summarizes three distinct mediating pathways through which social value orientation attenuates aggressive behavior, along with statistical tests comparing the magnitudes of these indirect effects. The total indirect effect is statistically significant, as indicated by a Bootstrap 95% confidence interval that excludes zero (-0.044; 95% CI [−0.058, −0.031]), accounting for 14.342% of the total effect. Specifically: (1) the indirect effect via perspective taking (social value orientation → perspective taking → aggressive behavior) is −0.028 (95% CI [−0.039, −0.018]); (2) the indirect effect via general trust (social value orientation → general trust → aggressive behavior) is −0.013 (95% CI [−0.021, −0.007]); and (3) the serial indirect effect operating through both perspective taking and general trust (social value orientation → perspective taking → general trust → aggressive behavior) is −0.003 (95% CI [−0.005, −0.001]). All three confidence intervals exclude zero, confirming the statistical significance of each pathway. Pairwise comparisons of the indirect effects further reveal that indirect effect 1 is significantly larger than indirect effects 2 and 3, and indirect effect 2 is significantly larger than indirect effect 3.

**TABLE 3:** The chain mediation effect of perspective taking and general trust.

Effect pathway	Indirect effect value	SE	95% *CI* LL	95% *CI* UL	Relative mediation effect
Total indirect effect	–0.044	0.007	–0.058	–0.032	14.342%
Indirect effect 1	–0.028	0.005	–0.039	–0.018	9.017%
Indirect effect 2	–0.013	0.001	–0.021	–0.007	4.378%
Indirect effect 3	–0.003	0.007	–0.005	–0.001	0.947%
Comparison 1	–0.014	0.007	–0.029	–0.001	
Comparison 2	–0.025	0.005	–0.036	–0.016	
Comparison 3	–0.011	0.003	–0.018	–0.005	

All the coefficients have been standardized. Comparison 1 and 2 respectively represent the differences between indirect effect 1 and 2, and between indirect effect 1 and 3. Comparison 3 represents the difference between indirect effect 2 and 3.

## 4 Discussion

The present study reveals a significant negative association between social value orientation and aggressive behavior among college students, such that higher levels of prosocial orientation correspond to lower frequencies of aggressive behavior. This finding aligns with prior empirical evidence (Cornelissen et al., 2011; Hilbig and Zettler, 2009) and provides support for Research Hypothesis 1. Drawing on the Ił theory of aggression (Finkel and Hall, 2018), which posits that the behavioral manifestation of aggression depends on the relative balance between inhibitory control and aggressive motivation, our results suggest that individuals with stronger prosocial orientations exhibit enhanced capacity to regulate impulsive aggressive tendencies – thereby reducing the likelihood and frequency of aggressive acts. Complementing this theoretical interpretation, extant literature further indicates that prosocial skill training serves as an effective intervention for mitigating aggression (Coyne et al., 2018), whereas antisocial tendencies constitute a robust risk factor for aggression (Jones et al., 2011). Collectively, these convergent findings reinforce the inhibitory role of social value orientation in modulating aggressive behavior.

Consistent with Hypothesis 2, the present findings demonstrate that perspective taking mediates the association between social value orientation and aggressive behavior among college students. Specifically, higher SVO scores are linked to greater perspective-taking capacity, a factor that coincides with fewer aggressive behavioral reports. Prior research indicates that individuals with a more prosocial SVO exhibit greater concern for others’ welfare, heightened sensitivity to others’ needs and viewpoints, and a stronger tendency to adopt others’ perspectives when interpreting social situations (Declerck and Bogaert, 2008; Derks et al., 2015; Dong et al., 2018). Furthermore, empirical evidence consistently reveals a robust negative association between perspective taking and aggression. Studies have shown that perspective taking interventions significantly attenuate aggressive responses in adolescent populations (Kahhale et al., 2024; Pozzoli et al., 2017). Extending these findings to a Chinese cultural context, the present study replicates and corroborates the hypothesized three-variable mediation model linking SVO, perspective taking, and aggression, thereby enhancing its cross-cultural generalizability. Collectively, these results support a partial mediation effect of perspective taking in the relationship between social value orientation and aggressive behavior among college students.

Furthermore, the present findings indicate that general trust partially mediates the association between social value orientation and aggressive behavior among college students, thereby supporting Hypothesis 3. Specifically, students exhibiting higher levels of prosocial social value orientation tend to report greater general trust, which in turn is associated with lower self-reported aggressive behavior. Prior research suggests that individuals with a stronger prosocial orientation demonstrate enhanced resilience to interpersonal risk, more positive expectations regarding social interactions, and greater willingness to engage in cooperative and trusting behaviors (Derks et al., 2014; Kanagaretnam et al., 2009). Concurrently, empirical evidence consistently demonstrates that higher levels of general trust are negatively associated with various forms of antisocial conduct, including aggression, bullying, and violent behavior (Rotenberg et al., 2021; Rotenberg and Fonseca, 2024; Yang et al., 2025). Collectively, these patterns support a partial mediation model wherein social value orientation exerts its influence on aggressive behavior both directly and indirectly via its positive association with general trust.

Finally, the present findings indicate that the sequential mediation pathway comprising perspective taking followed by general trust significantly accounts for the association between social value orientation and aggressive behavior among college students, thereby providing robust empirical support for Hypothesis 4. Prior scholarship consistently identifies both perspective taking and general trust as core prosocial capacities, with theoretical and empirical evidence underscoring their reciprocal interdependence (Evans and Krueger, 2011). Specifically, perspective taking aligns with stronger cognitive and affective understanding of others’ perspectives, a pattern linked to elevated interpersonal trust (Fett et al., 2014). Moreover, experimental studies demonstrate that targeted interventions to enhance perspective taking reliably increase levels of general trust (Erle et al., 2018; Kong et al., 2023). Collectively, these results suggest that social value orientation exerts an indirect influence on college students’ aggressive behavior through the serial mediating mechanism of perspective taking and subsequent general trust.

## 5 Implications and limitations

Our serial mediation model reveals a statistically significant direct link between social value orientation and college students’ aggressive behavior, alongside preliminary statistical evidence for the chain mediating roles of perspective taking and general trust in this association. Importantly, we acknowledge that despite reaching statistical significance, the magnitude of this chained indirect effect is small, so this chain mediating pathway only partially explains the association between prosocial value orientation and aggression. These results offer tentative insights into one psychological route through which prosocial tendencies may weakly buffer aggressive tendencies, carrying modest theoretical value for developmental and social psychology and limited auxiliary reference for campus anti-violence work. Interventions centered on nurturing prosocial values, improving perspective-taking skills, and boosting generalized trust may act as supplementary supportive strategies that correlate with marginally lower self-reported student aggression.

For educational practitioners, we propose several tentative auxiliary intervention directions. First, universities may modestly incorporate prosocial value cultivation into routine teaching and campus culture construction, including targeted political education courses, ethical thematic lectures, and experiential moral education activities. These activities may correspond to mild gains in prosocial tendencies; however, their statistical linkage to fewer aggressive behaviors is weak, consistent with our small mediation effect. Second, low-cost practices including role-play exercises and reflective group counseling may be implemented in schools; such activities tend to coincide with modest gains in students’ empathic cognition and interpersonal awareness. Third, moral education can integrate media literacy training and positive peer communication activities to moderately lift generalized trust and social adaptability. Taken together, these coordinated measures may deliver mild auxiliary benefits for mitigating student aggression, yet they must be matched with other targeted anti-bullying and conflict management interventions given the weak explanatory power of our focal serial mediation pathway.

This study, like many others in the field, is subject to several limitations. First, the data are cross-sectional, precluding robust inference regarding temporal dynamics or causal relationships among the variables under investigation. To address this, future research should adopt longitudinal designs, ideally integrating within-subject measurement protocols, to more rigorously examine developmental trajectories and causal mechanisms. Second, while aggressive behavior has increasingly migrated from traditional face-to-face contexts to online environments driven by the proliferation of digital communication platforms such as social media and mobile applications, the present study focuses exclusively on conventional forms of aggression and does not assess online aggression. Subsequent work should therefore extend the scope to investigate how social value orientations shape aggressive behavior among college students in virtual spaces. Third, the current sample is relatively narrow in both size and developmental range. Given that aggression often peaks and undergoes significant transformation during adolescence, future studies would benefit from a lifespan developmental perspective, examining not only college-aged individuals but also younger adolescents, to map the psychological development of aggression and elucidate how contextual, interpersonal, and intrapersonal factors differentially influence its expression across developmental stage. Fourth, the chained indirect effect identified here carries only a small effect size. While statistically significant, this sequential mediating pathway explains limited variance in aggressive behavior, meaning perspective taking and general trust together constitute a relatively weak intermediate mechanism linking social value orientation to aggression. This constraint restricts both the theoretical explanatory power and the practical intervention utility of our findings.

## 6 Conclusions

This study explores how social value orientation correlates with aggressive behavior among Chinese college students based on social and developmental psychological theories, testing both its direct association and the serial indirect pathways via perspective taking and general trust. Statistical results show prosocial social value orientation significantly and negatively predicts aggressive behavior. Statistically significant serial mediation also emerges: prosocial orientation correlates with stronger perspective-taking, which correlates with higher general trust, and trust in turn correlates with lower aggression. Nevertheless, we stress that this sequential indirect effect is small in magnitude. Although detectable statistically, this dual mediating chain exerts only a weak substantive influence on aggressive behavior, and the insights derived from this pathway should be interpreted with appropriate caution in subsequent theoretical extension and practical intervention design.

## Data Availability

The raw data supporting the conclusions of this article will be made available by the authors, without undue reservation.
